# SIRT2 Alleviates Chronic Cold Stress-Induced Lung Injury by Regulating Lung Macrophage M1 Polarization

**DOI:** 10.3390/cimb48060543

**Published:** 2026-05-22

**Authors:** Bin Xu, Shizhen Lu, Rongge Xia, Qi Han, Zhiqi Zhu, Xinpeng Chen, Huiying Shi, Wencong Wu, Wanqun Xing, Jingjing Lu

**Affiliations:** College of Animal Science and Veterinary Medicine, Heilongjiang Bayi Agricultural University, Daqing 163319, China; xubin@byau.edu.cn (B.X.);

**Keywords:** SIRT2, chronic cold stimulation, alveolar macrophages, inflammation, oxidative stress, polarization

## Abstract

SIRT2 (Sirtuin 2) is an NAD+-dependent deacetylase that exerts crucial regulatory effects on immune homeostasis and macrophage activation. While chronic cold exposure is a known predisposing factor for pulmonary dysfunction, the precise mechanisms by which SIRT2 potentially modulates lung macrophage polarization under cold stress remains poorly understood. In this study, we evaluated the protective capacity of SIRT2 using both wild-type (WT) and *Sirt2*-knockout (*Sirt2*−/−) murine models subjected to chronic cold exposure (4 °C for 3 h daily over 21 days). Our results demonstrated that *Sirt2* deficiency significantly exacerbated cold-induced pulmonary histopathological damage and increased the secretion of pro-inflammatory cytokines (TNF-α, IL-1β, and IL-6) (*p* < 0.05). Furthermore, chronic cold stress triggered a macrophage-centered inflammatory response, a process wherein SIRT2 was found to curtail M1 pro-inflammatory polarization. To further investigate these mechanisms, in vitro experiments were conducted using the mouse alveolar macrophage cell line MH-S. While LPS was utilized as a canonical inflammatory stimulus to mimic the injury environment, SIRT2 overexpression was found to reverse the LPS-induced increase in M1 markers and attenuate inflammatory cytokine secretion. These findings suggest that SIRT2 maintains intracellular homeostasis by modulating macrophage plasticity and plays a protective role in the development of chronic cold stimulus-induced lung injury. Consequently, SIRT2 activation may represent a potential therapeutic pathway for the treatment of environment-related respiratory diseases.

## 1. Introduction

The association of low ambient temperatures with susceptibility to respiratory infections is generally accepted, and low temperatures are seen as the most important environmental factor regulating the survival, stability and spread of pathogens [1]. Cold temperatures have been reported to have a negative impact on the body’s immunity, leading to an increased incidence of other respiratory diseases such as viral influenza, diarrhea, asthma and pneumonia [2,3,4,5]. In severe cases, low ambient temperatures can even increase the risk of death in patients with lung disease. Therefore, it is necessary to investigate the relationship between cold stimulation and the severity of lung injury.

Macrophages are the most important innate immune cells and the first line of defense against viruses and bacteria [3]. As highly plastic cells, their maintenance of homeostasis is related to their activation phenotypes, and they respond to different pathophysiological conditions and the surrounding microenvironment with specific phenotypic and functional responses [4]. Macrophage activation exists on a continuum in response to microenvironmental cues; however, for simplified functional characterization, macrophages are often broadly categorized into two extremes: classically activated macrophages (M1) and alternatively activated macrophages (M2). M1 macrophages are pivotal in the pro-inflammatory response of the host defense, whereas M2 macrophages contribute to the anti-inflammatory response and tissue remodeling. There is increasing evidence that macrophage polarization is closely associated with the development of inflammatory lung diseases such as acute lung injury (ALI), acute respiratory distress syndrome (ARDS), allergic asthma and chronic obstructive pulmonary disease (COPD) [5,6,7]. The balance between M1 and M2 phenotypes in macrophages determines the fate of an organ during inflammation or injury, and sustained M1 polarization can lead to the release of tumor necrosis factor alpha (TNF-α), interleukin 1 (IL-1), nitric oxide (NO) and reactive oxygen species (ROS), thereby triggering severe inflammatory responses. In contrast, M2 macrophages express inflammatory inhibitory factors that exert an inhibitory effect on the inflammatory response and tissue repair. Alveolar macrophages (AMs), located on the surface of the alveolar interstitial space as an essential subpopulation of resident immune cells, are crucial for maintaining the dynamic balance of lung immunity. They play a critical role in the initiation, resolution and tissue repair processes associated with lung inflammation [8,9]. Previous studies have found that cold stimulation inhibits phagocytosis in AMs [10,11,12,13]. Therefore, it is particularly important to understand the relationship between cold stimulus-induced lung injury and polarization of AMs. Chronic cold exposure is not only a physical stressor but also a trigger for systemic inflammatory responses, leading to compromised pulmonary barrier function.

Sirtuin2 (SIRT2) is a NAD+-dependent class I histone deacetylase that shuttles between nucleoplasmic compartments and plays a role in tumorigenesis, neuroprotection, glycolipid metabolism and cell cycle progression [14,15,16,17,18]. SIRT2 deacetylates different substrates and thus regulates important cellular metabolic pathways. Studies have demonstrated that SIRT2 can prevent endoplasmic reticulum stress and facilitate the repair of intestinal barrier damage through the regulation of FOXO1 [19]. In terms of inflammatory processes, SIRT2 was shown to deacetylate NF-κB p65, thereby attenuating the inflammatory response, renal tubular inflammation and ischemia-reperfusion-induced hepatocyte inflammation in a model of brain injury [20,21,22,23]. In our previous experiments, SIRT2 expression changed during cold stress and was involved in the cold stimulation process [24,25]. Studies have demonstrated that SIRT2 can regulate macrophage fate and its inflammatory phenotype [26]. Recent studies have highlighted SIRT2 as a metabolic sensor that deacetylates key transcription factors like NF-κB p65, thereby curbing excessive inflammation. However, whether SIRT2 manages the pulmonary immune landscape under low-temperature stress remains an unexplored frontier.

Given the “fate-determining” role of SIRT2 in macrophages across diverse metabolic microenvironments, this study aimed to investigate whether SIRT2 activity contributes positively to the resolution of cold-stimulation-induced lung injury by modulating the phenotype of alveolar macrophages.

## 2. Materials and Methods

### 2.1. Animal Experimental Models

Six-to-eight-week-old male specific-pathogen-free (SPF) wild-type (WT) C57BL/6J mice and *Sirt2* knockout (*Sirt2*−/−) mice on a C57BL/6J background were purchased from Cyagen Biosciences Co., Ltd. (Suzhou, Jiangsu, China). Male mice were exclusively selected to avoid the potential confounding effects of female hormonal cycles on systemic inflammatory and oxidative stress responses. *Sirt2*−/− mice were constructed using CRISPR-Cas9 technology and purchased from Cyagen Biosciences. The experiment was divided into four groups, Wild-type room temperature group (WT-Control), *Sirt2* knockout room temperature group (KO-Control), Wild-type cold stimulation group (WT-Cold) and *Sirt2* knockout cold stimulation group (KO-Cold), with 15 mice in each group. A total of 60 male mice (*n* = 15 per group) were allocated to ensure sufficient tissue availability for multiple downstream applications, including histopathology, Western blotting and qPCR. For each specific assay, *n* = 3–5 independent biological replicates were randomly selected from the cohort to ensure statistical stringency and experimental reproducibility. This sample size was determined based on preliminary studies and a power analysis to detect significant biological differences in protein and gene expression levels. For the chronic cold stimulation model, mice were placed in a 4 °C climatic chamber for 3 h per day (at non-fixed times between 8:00 a.m. and 8:00 p.m.) and then returned to room temperature (24 ± 2 °C) for the remainder of the day. This daily procedure was repeated for 3 consecutive weeks. Mice were placed in a temperature/humidity-controlled environment (24 ± 2 °C/40%) and were maintained on a 12 h light/12 h dark cycle with free access to food and water. All experimental procedures were approved by the Management Committee of Laboratory Animal Center of Heilongjiang Bayi Agricultural University.

### 2.2. Cell Culture and Treatment

The mouse alveolar macrophage cell line, MH-S (Fenghui) was cultured in 1640 medium (Gibco; Thermo Fisher Scientific, Inc., Waltham, MA, USA; Cat. No. C11995500BT) supplemented with 10% fetal bovine serum (FBS; Gibco; Thermo Fisher Scientific, Inc., Waltham, MA, USA; Cat. No. #10099) and 1% penicillin/streptomycin (Beijing Solarbio Science & Technology Co., Ltd., Beijing, China; Cat. No. P1400) at 37 °C and 5% CO_2_. Cell growth was observed daily, with the culture medium replaced regularly. When the cells reached 80% fusion, they were inoculated in Petri dishes. For the LPS (Beyotime Biotechnology Co., Ltd., Shanghai, China; Cat. No. ST1470) induced M1 pro-inflammatory polarized cell model, the cells were treated for 6 h with 100 ng/mL LPS. To explore the effect of SIRT2 high/low expression efficiency on the polarization of MH-S cells, Lipo8000 (Beyotime Biotechnology Co., Ltd., Shanghai, China; Cat. No. C0533) was used to transfect the SIRT2 adenoviruses Flag-SIRT2 and sh-SIRT2 (Syngentech) for 48 h, followed by LPS treatment for 6 h before sample collection.

### 2.3. Hematoxylin–Eosin (H&E) Staining

Fresh lung tissues were taken from mice after euthanasia and fixed with 4% paraformaldehyde (Biosharp Life Sciences; Ako Bio Co., Ltd., Hefei, Anhui, China; Cat. No. #BL1515A). After paraffin embedding, 4-μm-thick sections were cut for H&E staining and observed under a light microscope, thereby assessing the severity of lung injury.

Lung injury was semi-quantitatively assessed using a modified scoring system based on established protocols. For each section, five to ten random fields were evaluated under high-power magnification. The scoring criteria included: (i) alveolar septal thickening, (ii) inflammatory cell infiltration (primarily neutrophils and macrophages) and (iii) structural congestion or hemorrhage. Each parameter was graded on a scale of 0 to 4:

Grade 0: Normal lung morphology.

Grade 1: Minimal damage (affecting <25% of the field).

Grade 2: Mild-to-moderate damage (25%–50% of the field).

Grade 3: Moderate-to-severe damage (50%–75% of the field).

Grade 4: Severe damage or total loss of alveolar architecture (>75% of the field).

### 2.4. Detection of MDA and GSH

Mouse lung tissue malondialdehyde (MDA) and glutathione (GSH) were selected to be detected by the kit, and the specific assay method was carried out by referring to the instruction of the kit (Beyotime, #S0131S, #S0053).

### 2.5. Western Blot Analysis

To extract total proteins, lung tissues were mechanically homogenized in RIPA lysis buffer containing protease inhibitors (Beyotime Biotechnology Co., Ltd., Shanghai, China; Cat. No. P0013B). After a 30-min incubation on ice, the lysates were centrifuged at 12,000 rpm for 15 min at 4 °C, and the supernatants were collected. Protein concentrations were quantified via an Enhanced BCA Protein Assay Kit (Beyotime Biotechnology Co., Ltd., Shanghai, China; Cat. No. P0010). Equal amounts of protein (30 μg) were separated by 10% SDS-PAGE and electrotransferred onto polyvinylidene fluoride (PVDF) membranes (Merck Millipore, Burlington, MA, USA). The membranes were then blocked with 5% non-fat dry milk at room temperature for 2 h, followed by three 5-min washes with 1 × TBST (20 mmol/L Tris-HCl, pH 7.5, 150 mmol/L NaCl, 0.05% Tween 20). They were then incubated overnight at 4 °C with the following primary antibodies: SIRT2 (19655-1-AP, Proteintech, 1:5000), IL-6 (66146-1-Ig, Proteintech, 1:1000), IL-1β (16806-1-AP, Proteintech, 1:2000), TNF-α (17590-1-AP, Proteintech, 1:1000), HSP60 (15282-1-AP, Proteintech, 1:2000), HSP70 (10995-1-AP, Proteintech, 1:5000), HSP90 (13171-1-AP, Proteintech, 1:2000), CAT (21260-1-AP, Proteintech, 1:3000), SOD1 (10269-1-AP, Proteintech, 1:5000), Keap1 (10503-2-AP, Proteintech, 1:2000), HO-1 (10701-1-AP, Proteintech, 1:1000), Nrf2 (#12721, Cell Signaling Technology, Danvers, MA, USA, 1:1000) and β-Actin (66009-1-Ig, Proteintech, 1:20,000). The next day, after washing the membrane five times with 1 × TBST, the membrane was incubated with HRP-conjugated Affinipure goat anti-mouse IgG (H + L) (SA00001-1, Proteintech, 1:10,000) or HRP-conjugated Affinipure goat anti-rabbit IgG (H + L) (SA00001-1, Proteintech, 1:10,000) as the secondary antibody for 1 h at room temperature. Protein expression was visualized by using the ECL Chemical Discharge Kit (Vazyme, Nanjing, China, #E422-01) and the intensity of each protein expression was detected by Image Lab software (6.0.1). The protein expression level was expressed as the ratio of the optical density of each target protein to the optical density of β-Actin.

### 2.6. Quantitative Real-Time PCR

Total RNA was extracted using TRIzol reagent (Invitrogen; Thermo Fisher Scientific, Inc., Carlsbad, CA, USA; Cat. No. 15596018CN) and total RNA purity was determined by confirming that the OD260/0D280 ratio was between 1.8 and 2.0. cDNA was synthesized by reverse transcription of 5 μg of total RNA using the Transcriptor First Strand cDNA Synthesis Kit (Takara Bio Inc., Kusatsu, Shiga, Japan, RR047A). The synthesized cDNA was subsequently used for quantitative real-time PCR (qPCR) with SYBR Green (Takara Bio Inc., Kusatsu, Shiga, Japan, RR420A) and the following primers:

IL-6 (Mouse):

Forward 5′-AGA GAC TTC CAG CCA GTT GC-3′

Reverse 5′-AGT CTC CTC TCC GGA CTT GT-3′;

IL-1β (Mouse):

Forward 5′-TCG CAG CAG CAC ATC AAC AAG AG-3′

Reverse 5′-TGC TCA TGT CCT CAT CCT GGA AGG-3′;

TNF-α (Mouse):

Forward 5′-GCG ACG TGG AAC TGG CAG AAG-3′

Reverse 5′-GCC ACA AGC AGG AAT GAG AAG AGG-3′;

CD11b (Mouse):

Forward 5′-CCA TGA CCT TCC AAG AGA ATG C-3′

Reverse 5′-ACC GGC TTG TGC TGT AGT C-3′;

IBA1 (Mouse):

Forward 5′-CCC TCT GAT GTG GTC TGC AC-3′

Reverse 5′-GAC TTT CCC CGG GAT GGA AG-3′;

β-actin (Mouse):

Forward 5′-TAT GCT CTC CCT CAC GCC ATC C-3′

Reverse 5′-GTC ACG CAC GAT TTC CCT CTC AG-3′.

mRNA expression levels were analyzed by the 2^−ΔΔCT^ method, normalized to β-Actin as an internal reference gene.

### 2.7. Immunofluorescence

MH-S cells in good growth condition were inoculated into 24-well plates containing cell coverslips, and the cells were treated according to the above conditions. The cells were fixed with pre-cooled 4% paraformaldehyde for 15 min, washed three times with 1 × PBST (Sangon Biotech, Shanghai, China; Cat. No. C006162), and then permeabilized with 0.2% TritonX-100 (Beyotime, #P0096) for 15 min. The cells were blocked with 5% BSA (Solarbio, #PC0001) in PBS for 1 h at room temperature, washed five times with 1 × PBST and incubated with the corresponding primary antibodies (F4/80 (BioLegend, San Diego, CA, USA; Cat. No. 123122), iNOS (Proteintech, Chicago, IL, USA; Cat. No. 18985-1-AP)) overnight at 4 °C. The next day, after washing, add the corresponding fluorescein-coupled secondary antibody Alexa Fluor 488 labeled goat anti-rabbit IgG (H + L) (Beyotime Biotechnology Co., Ltd., Shanghai, China; Cat. No. RGAR002) or Alexa Fluor 647 labeled goat anti-mouse IgG (H + L) (Beyotime Biotechnology Co., Ltd., Shanghai, China; Cat. No. RGAM005). Incubation was carried out for 1 h at room temperature away from light. After removing the secondary antibody, the crawler was inverted onto a slide with a drop of anti-fluorescence burst sealer (Beyotime Biotechnology Co., Ltd., Shanghai, China; Cat. No. P0131) containing 4′,6-diamidino-2-phenylindole (DAPI) using forceps. Staining results were observed under an EVOS f1 fluorescence microscope.

### 2.8. Statistical Analysis

GraphPad Prism 8.0 software was used to calculate all statistical parameters, and differences between the two groups were statistically analyzed by *t*-test and the rest by Two-way ANOVA followed by Tukey’s post-hoc test for multiple comparisons. *p* < 0.05 was considered statistically significant and data are expressed as mean ± SD.

## 3. Results

### 3.1. Effects of Sirt2 Deficiency on Mouse Lung Histology Under Chronic Cold Stimulation

**Figure 1:**
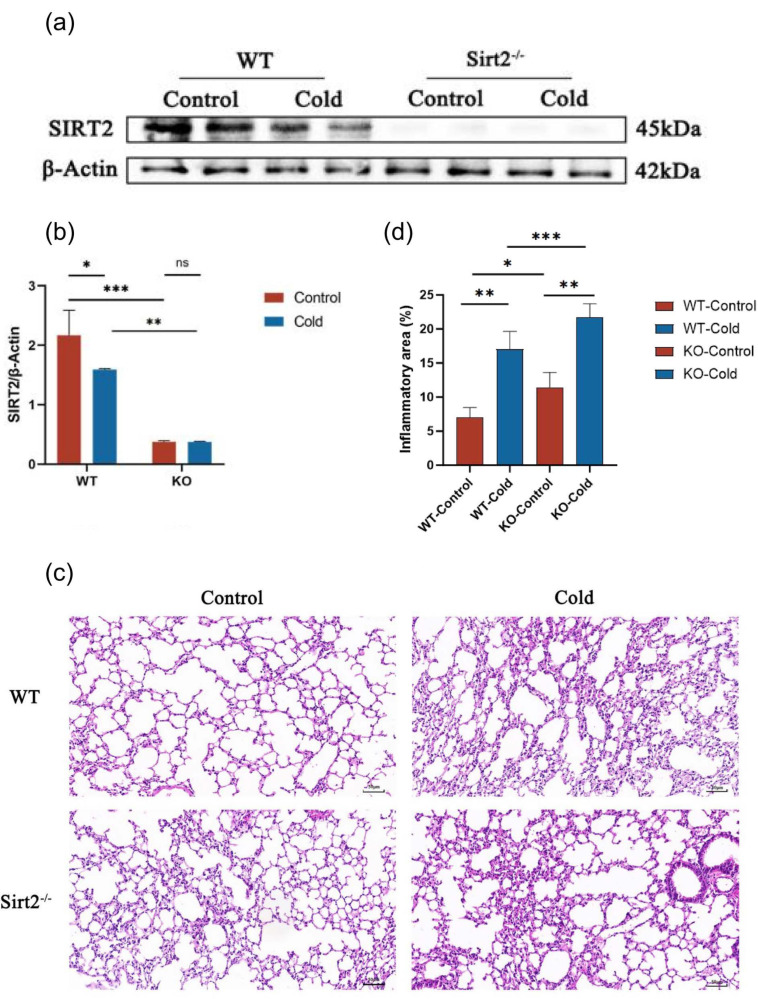
*Sirt2* deficiency exacerbates chronic cold stimulation-induced structural damage in mouse lung tissue. (**a**) Western blot detection of SIRT2 protein expression levels in the lungs of WT and *Sirt2*−/− mice. (**b**) Correlative quantitative analysis of SIRT2 (/β-Actin). (**c**) H&E staining of WT and *Sirt2*−/− mice lung sections. (Scale bar, 50 μm). (**d**) Quantitative analysis of the lung inflammatory area. Data are reported as mean ± SD *n* = 4. * *p* < 0.05, ** *p* < 0.01, *** *p* < 0.001, ns indicates insignificant differences. WT, wild type; SIRT2, sirtuin 2; H&E, hematoxylin−eosin.

To investigate the relationship between *Sirt2* gene deletion and chronic cold stimulation-induced lung injury in mice, a model of chronic cold stimulation at 4 °C for 3 h/d for 3 weeks was developed using wild-type (WT) mice and *Sirt2*−/− mice. The results of SIRT2 expression assay in the lung tissues of mice in each group showed that SIRT2 protein was not expressed in the lung tissues of *Sirt2*−/− mice. Furthermore, chronic cold stimulation significantly reduced the protein expression level of SIRT2 specifically in the lung tissues of WT mice, as it remained completely undetected in *Sirt2*−/− mice (Figure 1a,b). H&E staining of lung tissue revealed that the WT-Cold group experienced significant inflammatory cell infiltration and slight alveolar structure damage compared to the WT-Control group. The lungs of mice in the KO-Control group showed slight tissue damage, whereas the lung tissues of mice in the KO-Cold group showed more severe damage, as evidenced by severe damage to alveolar structure, thickening of alveolar walls, hemorrhage in alveoli and interstitium, and increased recruitment of inflammatory cells (Figure 1c). Histopathological examination revealed varying degrees of pulmonary architectural disruption across the experimental groups. Histopathological evaluation confirmed that cold stress induced varying degrees of pulmonary impairment across different genotypes. As shown in Supplementary Table S1, the WT-Cold group exhibited significant inflammatory cell infiltration and mild disruption of alveolar architecture compared to the WT-Control group. While KO-Control mice showed only minimal pulmonary changes, the most severe lung injury was observed in the KO-Cold group. In this group, the alveolar structure was profoundly damaged, characterized by marked septal thickening, extensive alveolar and interstitial hemorrhage and a substantial increase in inflammatory cell recruitment. Quantitatively, the lung injury score (LIS) escalated from 0.7 in the WT-Control group to 3.7 in the KO-Cold group, highlighting that the deficiency of *Sirt2* significantly exacerbates cold-induced lung injury. The above results suggest that *Sirt2* deficiency aggravates structural lung tissue damage in cold-stimulated mice.

### 3.2. Sirt2 Deficiency Exacerbates the Release of Inflammatory Mediators from Lung Tissue of Mice with Chronic Cold Stimulation

**Figure 2:**
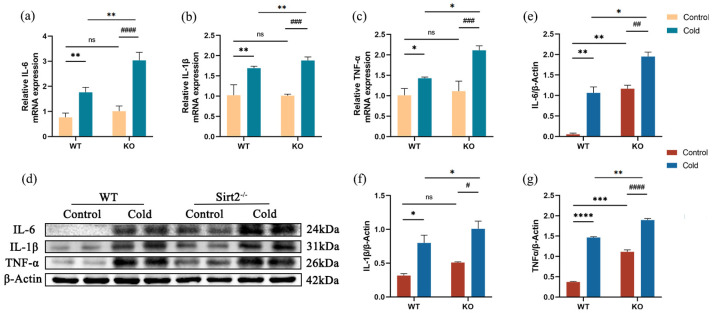
*Sirt2* deficiency exacerbates the release of inflammatory mediators from lung tissues of chronically cold-stimulated mice. (**a**–**c**) Detection of mRNA expression levels of IL-6, IL-1β, and TNF-α in the lungs of WT and *Sirt2*−/− mice by qRT-PCR. (**d**) Expression levels of inflammation-related proteins in the lungs of WT and *Sirt2*−/− mice detected by Western blot. (**e**–**g**) Quantitative analysis of IL-6, IL-1β and TNF-α correlation (/β-Actin). Data are reported as mean ± SD (*n* = 4). Data represent *n* = 4 independent biological replicates randomly selected from a larger cohort of 15 animals per group. * *p* < 0.05, ** *p* < 0.01, *** *p* < 0.001, **** *p* < 0.0001, significantly different from the WT-Control group. # *p* < 0.05, ## *p* < 0.01, ### *p* < 0.001, #### *p* < 0.0001, ns indicates insignificant differences, significantly different from the KO-Control group. IL-6, interleukin-6; IL-1β, interleukin-1 beta; TNF-α, Tumor necrosis factor-alpha.

Given that lung injury is linked to the release of intracellular inflammatory factors triggered by cold stimuli, it can lead to pulmonary vascular disorders and disruption of the alveolar–capillary barrier. This part explores the effect of chronic cold stimulation on the inflammatory response of the lungs under *Sirt2*-deficient conditions. The results of mRNA expression of pro-inflammatory cytokines in lung tissues showed that the mRNA expression levels of IL-6, IL-1β, and TNF-α in lung tissues of WT mice were significantly elevated after cold stimulation, *Sirt2*−/− mice exhibited a modest increase in inflammatory factor expression in the lungs compared to WT mice, which was further augmented under cold stimulation (Figure 2a–c). As expected, the protein expression levels of these proinflammatory factors were consistent with the mRNA expression levels, and higher proinflammatory cytokine expression was demonstrated within the lung tissues of *Sirt2*−/− mice under the influence of cold conditions (Figure 2d–g). Apparently, *Sirt2* deficiency is able to exacerbate the secretion of inflammatory factors in lung tissues of chronically cold-stimulated mice.

### 3.3. Sirt2 Deficiency Exacerbates Oxidative Stress Levels in Lung Tissues of Chronically Cold-Stimulated Mice

**Figure 3:**
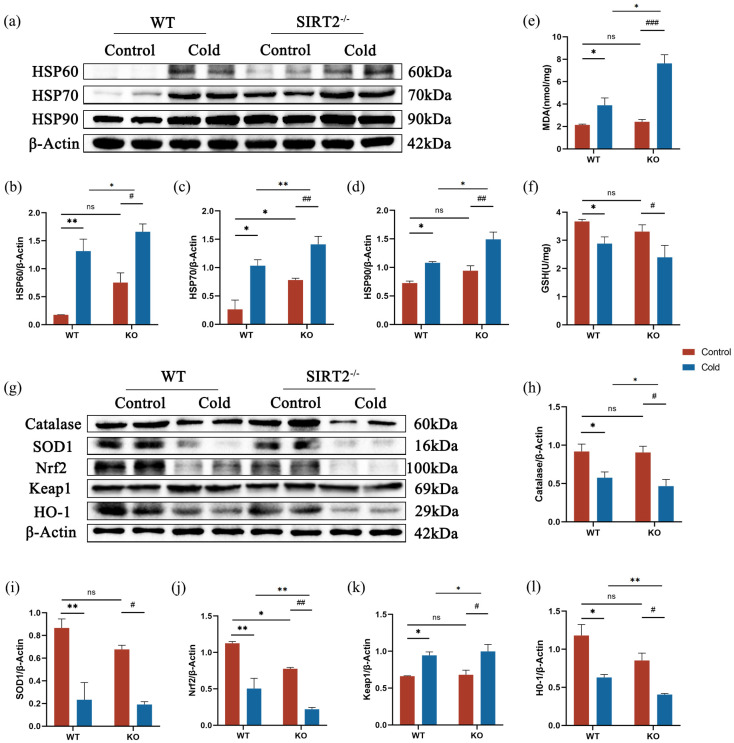
*Sirt2* deficiency exacerbates oxidative stress in lung tissues of chronically cold-stimulated mice. (**a**) Western blot detection of heat shock protein expression levels in the lungs of WT and *Sirt2*−/− mice. (**b**–**d**) Quantitative analysis of the correlation between HSP60, HSP70 and HSP90 (/β-Actin). (**e**,**f**) Determination of MDA and GSH in lung tissues of WT and *Sirt2*−/− mice by ELISA. (**g**) Expression levels of oxidative stress and anti-oxidative stress proteins in the lungs of WT and *Sirt2*−/− mice detected by Western blot. (**h**–**l**) Correlation quantification of Catalase, SOD1, Nrf2, Keap1 and HO-1 (/β-Actin). Data are reported as mean ± SD (*n* = 4). Data represent *n* = 4 independent biological replicates randomly selected from a larger cohort of 15 animals per group. Comparisons with the WT-Control group are indicated with the * symbol, * *p* < 0.05, ** *p* < 0.01. Comparisons with the KO-Control group are indicated by the # symbol, # *p* < 0.05, ## *p* < 0.01, ### *p* < 0.001, ns indicates insignificant differences. HSP60, Heat shock protein 60; HSP70, Heat shock protein 70; HSP90, Heat shock protein 90; MDA, Malondialdehyde; GSH, glutathione; CAT, Catalase; SOD1, Superoxide dismutase 1; Nrf2, Nuclear factor erythroid 2-related factor 2; Keap1, Kelch like ECH associated protein 1; HO-1, Heme Oxygenase-1.

Due to the higher concentrations of oxygen to which lung tissue is exposed compared to other organs, it is also more susceptible to damage from oxidative stress. In the present study, we further observed whether chronic cold stimulation under *Sirt2*-deficient conditions exacerbates oxidative lung injury. Heat shock protein is an important indicator of whether an animal is under stress. The results of the expression of heat shock proteins in the lungs of mice in each group showed that the expression levels of HSP60, HSP70 and HSP90 in the lungs of WT mice were significantly elevated after cold stimulation, while the protein expression of only HSP70 was slightly elevated in the lungs of *Sirt2*−/− mice; however, the elevation of the expression level of heat shock proteins was more significantly elevated in the lungs of *Sirt2*−/− mice under the cold stimulation condition (Figure 3a–d). The results of oxidative stress index assay showed that the MDA content in the lungs of WT mice was elevated and GSH activity was decreased after cold stimulation, and the changes of MDA and GSH in the lungs of *Sirt2*−/− mice were not significant; however, the MDA content in the lungs of *Sirt2*−/− mice was highly significantly elevated and the GSH activity was lower compared to that of WT mice after cold stimulation (Figure 3e,f). This result suggests that *Sirt2* deficiency under chronic cold stimulation can induce increased oxidative stress injury in mouse lungs. To further determine the effect of SIRT2 deficiency on the antioxidant capacity of lungs from cold-stimulated mice, the study examined the expression levels of the antioxidant enzymes CAT and SOD1. The results demonstrated that cold stimulation significantly decreased the expression levels of CAT and SOD1 in the lungs of WT mice. Similarly, the expression of CAT and SOD1 in the lungs of *Sirt2*−/− mice was significantly reduced following cold stimulation, with the decrease being more pronounced compared to WT mice (Figure 3g–i). The Keap1-Nrf2 signaling pathway is crucial for regulating intracellular stress responses and is significant in the pathogenesis of lung injury. The results showed that Keap1 expression was elevated and Nrf2 and HO-1 expression was decreased in the lungs of WT mice after cold stimulation, whereas *Sirt2*−/− mice exhibited more pronounced Keap1 up-regulation and Nrf2 and HO-1 down-regulation compared to WT mice under cold stimulation conditions (Figure 3j–l). The above results suggest that chronic cold stimulation under *Sirt2*-deficient conditions exacerbates oxidative stress injury and further inhibits antioxidant capacity in mouse lungs.

### 3.4. Sirt2 Deficiency Exacerbates the Pro-Inflammatory Polarization of Mouse Lung Macrophages in Response to Cold Stimulation

**Figure 4:**
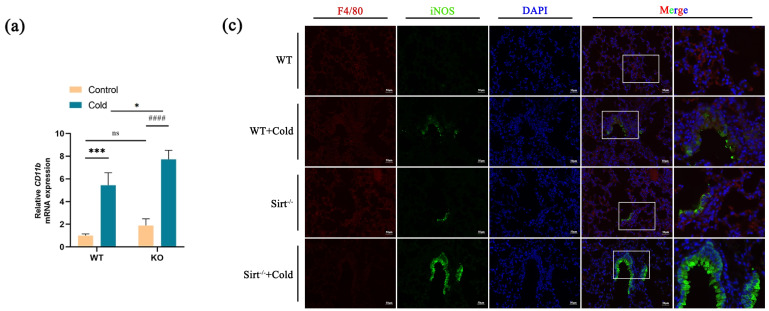

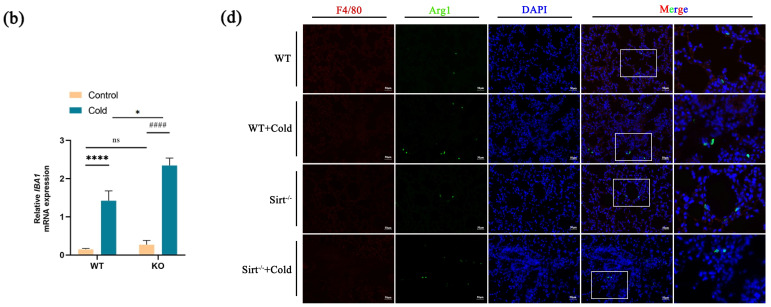
*Sirt2* deficiency exacerbates pro-inflammatory polarization in cold-stimulated mice lung macrophages. (**a**,**b**) Detection of mRNA expression levels of *CD11b* and *IBA1* in the lungs of WT and *Sirt2*−/− mice by qRT-PCR. (**c**) Immunofluorescence observation of the effect of *Sirt2* deletion on iNOS expression. (**d**) Immunofluorescence observation of the effect of *Sirt2* deletion on Arg1 expression (scale bar, 50 μm). Data are reported as mean ± SD (*n* = 3). Data represent *n* = 3 independent biological replicates randomly selected from a larger cohort of 15 animals per group. Comparisons with the WT-Control group are indicated with the * symbol, * *p* < 0.05, *** *p* < 0.001, **** *p* < 0.0001, ns indicates insignificant differences. Comparisons with the KO-Control group are indicated by the # symbol, #### *p* < 0.0001, ns indicates insignificant differences. CD11b, CD11 antigen-like family member B; IBA1, Ionized calcium binding adaptor molecule 1; F4/80, Mouse EGF-like module-containing mucin-like hormone receptor-like 1; iNOS, Inducible nitric oxide synthase; DAPI, 4′,6-diamidino-2-phenylindole; Arg1, Arginase 1.

Macrophage populations in the lungs maintain pulmonary homeostasis by phagocytosing inhaled particles and foreign pathogens, inducing cytokine production and antigen presentation, and facilitating clearance of particulate antigens. As an important component of the body’s natural immune system, macrophages mediate the development of various immunopathologies during inflammation and can be activated in response to the stimulation of a wide range of pathogens and are involved in the pathogenesis of diseases such as ALI/ARDS, whereas macrophage activation is the process by which they exhibit different functional phenotypes in response to the microenvironment. Therefore, we first examined the gene expression levels of macrophage activation markers CD11b (Figure 4a) and IBA1 (Figure 4b) by RT-qPCR, and the gene expression of lung macrophage activation markers was significantly elevated in WT mice under chronic cold stimulation conditions, and the KO-Cold group was more significantly elevated compared to the WT-Cold group, suggesting that the absence of *Sirt2* promotes lung macrophage activation induced by chronic cold stimulation. Next, the study examined lung macrophage M1 (F4/80, iNOS)/M2 (F4/80, Arg-1) phenotypic markers by immunofluorescence. The results showed that both WT mice and *Sirt2*−/− mice had a small amount of Arg-1 expression in lung macrophages after cold stimulation (Figure 4d), whereas *Sirt2*−/− mice showed a significant increase in iNOS expression in lung macrophages after cold stimulation (Figure 4c). This indicated that *Sirt2* deficiency enhances the activation of chronic cold-stimulated mouse lung macrophages, biasing them towards the M1 phenotype.

### 3.5. SIRT2 Inhibits LPS-Induced M1 Polarization in Alveolar Macrophages

**Figure 5:**
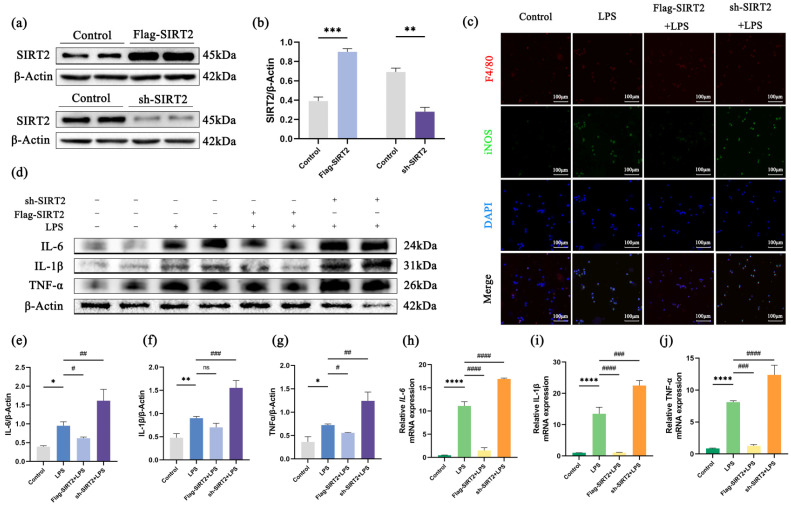
SIRT2 inhibits LPS-induced M1-type polarization in lung macrophages. (**a**) Flag-SIRT2 overexpression adenovirus infection and sh-SIRT2 interference plasmid transfection followed by Western blot detection of SIRT2 expression in MH-S cells. (**b**) Correlative quantitative analysis of SIRT2 (/β-Actin). (**c**) Immunofluorescence results of M1 phenotype (F4/80, iNOS) of MH-S cells under different treatments (scale bar, 100 μm). (**d**) Western blot detection of the expression level of inflammation-related proteins in MH-S cells under different treatments. (**e**–**g**) Quantitative analysis of IL-6, IL-1β and TNF-*α* correlation (/β-Actin), (**h**–**j**) Determination of mRNA expression levels of *IL-6*, *IL-1β*, and *TNF-α* in MH-S cells by qRT-PCR under different treatments. Data are reported as mean ± SD (*n* = 4). Data represent *n* = 4 independent biological replicates randomly selected from a larger cohort of 15 animals per group. Comparisons with the Control group are indicated with the * symbol, * *p* < 0.05, ** *p* < 0.01, *** *p* < 0.001, **** *p* < 0.0001. Comparisons with the LPS group are indicated by the # symbol, # *p* < 0.05, ## *p* < 0.01, ### *p* < 0.001, #### *p* < 0.0001, ns indicates insignificant differences. SIRT2, sirtuin 2; LPS, lipopolysaccharides; IL-6, interleukin-6; IL-1β, interleukin-1 beta; TNF-α, Tumor necrosis factor-alpha.

AMs, as a core component of macrophages in the lung, are the main innate immune effector cells in the lung and are highly plastic. The alveolar macrophage cell line MH-S was selected for the study, and 100 ng/mL LPS (an inducer of macrophage M1-type activation) was added to investigate whether SIRT2 is involved in influencing the phenotypic changes of AMs at the in vitro level, which in turn potentially modulates inflammatory responses. Studies were conducted to construct high-expressing adenovirus (Flag-SIRT2) and interfering plasmid (sh-SIRT2), and the transfection efficiency was determined by the protein expression of SIRT2 (Figure 5a,b). Immunofluorescence was used to detect macrophage M1-type markers (F4/80/iNOS). The results showed that SIRT2 overexpression significantly reduced the fluorescence expression intensity of iNOS compared with the LPS group. In contrast, knockdown of SIRT2 enhanced the fluorescence expression intensity of iNOS (Figure 5c). These data suggest that SIRT2 attenuates LPS-induced M1-type polarization of AMs. Subsequently, mRNA and protein expression of inflammatory cytokines were examined in the cells of each group. The results showed that both mRNA and protein expression levels of inflammatory cytokines were significantly higher compared to the control group; furthermore, the expression levels of inflammatory cytokines were both significantly lower after overexpression of SIRT2 under the influence of LPS, whereas the expression of inflammatory cytokines was higher after inhibition of SIRT2 expression compared to the LPS group (Figure 5d–j). The results suggest that SIRT2 inhibits the polarization of AMs toward M1-type, which in turn attenuates the inflammatory response.

## 4. Discussion

**Figure 6:**
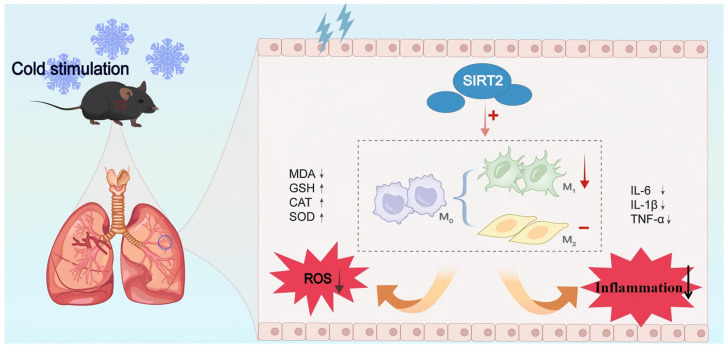
Chronic cold stimulus can cause some damage to lung tissue, and SIRT2 attenuates lung inflammation and oxidative stress induced by chronic cold stimulation by inhibiting M1-type polarization of lung macrophages. These findings provide a scientific basis for the prevention and treatment of respiratory diseases. SIRT2, sirtuin 2; ROS, Reactive oxygen species; MDA, Malondialdehyde; GSH, glutathione; CAT, Catalase; SOD, Superoxide dismutase; IL-6, interleukin-6; IL-1β, interleukin-1 beta; TNF-α, Tumor necrosis factor-alpha.

In this study, we demonstrated the protective role of SIRT2 in lung injury induced by chronic cold stimulation conditions and revealed the damage of cold stimulation on lung tissues, filling the functional area of SIRT2 research (Figure 6). Using *Sirt2* knockout (*Sirt2*−/−) mice as the first entry point, this study was the first to investigate cold, SIRT2, and lung injury, and tested whether SIRT2 plays a role in regulating macrophage phenotypes, which in turn affects the development of lung injury, providing a scientific basis for the prevention and treatment of respiratory diseases.

The physiology of the lungs is such that they are often exposed to viruses and bacteria present in the air and blood. The temperature plays a crucial role in regulating the host’s intrinsic, innate and adaptive immune response to respiratory infections [5,27,28,29]. In cryogenic environmental conditions, where cold outside air comes into contact with an organism’s alveolar walls, lung tissue can become persistently or excessively responsive to cryogenic exposure, leading to a high prevalence of respiratory disease and suppression of immune function. It has been shown that ambient hypothermia induces lung injury in mice, characterized by damage to the alveolar–capillary membrane and subsequent infiltration of inflammatory cells being the primary pathological feature [22,30,31]. It was observed that cold stimulation induced a mild inflammatory state with an increase in inflammatory cell recruitment. There was mild damage to lung histology in *Sirt2*−/− mice. Several reasons account for the visible weak injury observed in mice with *Sirt2* knockout under basal conditions. Firstly, SIRT2 belongs to the Sirtuins family, which includes seven isoforms (SIRT1-SIRT7). Therefore, the loss of one member’s function may activate the expression of other members to compensate similarly for the loss of SIRT2’s function. However, the ability of these other members to compensate is limited [32]. Secondly, the continuous renewal of cells and their rapid proliferation, differentiation, and death cycles within the lungs ensure a rapid regeneration of individual cells. This dynamic cellular turnover introduces numerous checkpoints, and makes it unlikely that mutations in individual genes will play a large role in the underlying conditions. However, the histological scores of the lungs in *Sirt2*−/− mice were significantly exacerbated by a cold stimulus challenge, as evidenced by severe disruption of alveolar structure and the enlargement of alveolar walls. The results presented in this section underscore the significance of SIRT2 and suggest that it may play an enhanced regulatory role under the stressful conditions of cold stimulation.

Inflammation, as a self-defense response of the body’s innate immune system in response to both endogenous and exogenous stimuli, accompanies a wide range of respiratory diseases [33]. SIRT2 is predominantly located in the cytoplasm and mediates protein deacetylation modifications in various pathological models. Knockdown of *Sirt2* results in more severe clinical and histological manifestations [34,35,36]. Notably, in our study, *Sirt2*−/− mice showed a discrepancy between mRNA and protein levels of certain cytokines under basal conditions; while mRNA levels remained stable, protein expression was elevated. This suggests that SIRT2 may regulate these pro-inflammatory factors primarily at the post-translational level—perhaps by modulating protein stability or translational efficiency via deacetylation—rather than through direct transcriptional control. Following chronic cold stimulation, the release of IL-6, IL-1β and TNF-α was significantly increased at both mRNA and protein levels in *Sirt2*−/− lungs.

Furthermore, the lung histological damage in *Sirt2*−/− mice was significantly exacerbated by cold challenge, characterized by severe alveolar structural disruption and increased wall thickening. These results underscore that SIRT2 is essential for maintaining pulmonary homeostasis under stressful conditions. We further explored whether oxidative stress contributes to this process. The surge in MDA content and Heat Shock Protein (HSP) levels in cold-stimulated mice corroborated a state of oxidative imbalance, which was markedly worsened in the absence of SIRT2. Our data points toward the potential involvement of the Keap1-Nrf2/HO-1 signaling pathway. While we observed inhibited expression of antioxidant enzymes (GSH, CAT, SOD) and defense proteins (Nrf2, HO-1) following cold stress, the precise interaction between SIRT2 and Nrf2 requires further mechanistic validation. It is plausible that SIRT2-mediated deacetylation influences the nuclear translocation or stability of Nrf2, thereby bolstering the antioxidant response elements (ARE) signaling.

Macrophages are important inflammatory cells involved in the progression of lung injury, responding to environmental signals by presenting one of two functional phenotypes and differentiating to form pro-inflammatory M1 or anti-inflammatory M2 macrophages, whose different polarization phenotypes correlate with the severity of lung injury [37,38,39,40]. Recent studies have highlighted the pivotal role of SIRT2 in regulating macrophage metabolism and its inflammatory phenotype [41,42,43,44,45]. In our in vivo model, *Sirt2* deficiency exacerbated M1-type polarization in the lungs. To further investigate the cellular mechanism in vitro, we utilized LPS-stimulated MH-S cells. While LPS is a canonical TLR4 agonist and does not directly replicate the physical nature of cold stress, it serves as a robust model to simulate the downstream inflammatory environment observed in lung injury. Our in vitro results showed that SIRT2 knockdown amplified the M1 marker iNOS, while SIRT2 overexpression reversed the high expression of inflammatory factors. These findings, though centered on a lung macrophage-biased response, suggest that SIRT2 maintains pulmonary homeostasis by suppressing excessive M1 polarization.

The aim of this study was to investigate the effect of SIRT2 on lung injury in mice under cold stress. We show that inhibition of SIRT2 under conditions of chronic cold stimulation leads to a tendency for AMs to become M1-type polarized, exacerbating lung inflammation and oxidative stress, thereby disrupting the healthy environment of the lungs. However, there are limitations to the current study. First, our in vivo findings regarding macrophage polarization were primarily based on lung tissue analysis; future research should involve the direct isolation of primary alveolar macrophages to conclusively verify cell-specific mechanisms. Second, the sample sizes presented in some figures (e.g., *n* = 3–4) represent independent biological replicates carefully selected from our larger allocated cohort (*n* = 15 per group) to ensure statistical stringency and experimental reproducibility. Third, this study exclusively utilized male mice to avoid the potentially confounding effects of female hormonal cycles on inflammatory pathways; however, this approach limits the generalizability of our conclusions across sexes, and future studies should include female cohorts. Finally, while gene expression profiles suggest the involvement of the Keap1-Nrf2 axis, further direct mechanistic assays (such as Co-IP) are needed to confirm the protein-level interactions.

## 5. Conclusions

In conclusion, our study provides evidence that SIRT2 may serve as a protective factor in alleviating lung inflammation and oxidative stress induced by chronic cold stimuli. We highlighted a novel role for SIRT2 in mitigating pulmonary damage, likely by inhibiting the M1-type polarization of macrophages and enhancing antioxidant defenses. While our findings point toward the involvement of the Keap1-Nrf2/HO-1 pathway, this interpretation remains preliminary and requires further confirmation through direct protein-interaction studies. Given the limitations in cell-type specificity and sex-specific generalizability, these results offer a foundation for future research into SIRT2 as a potential therapeutic target for environmental stress-related respiratory conditions.

## Data Availability

The original contributions presented in this study are included in the article/Supplementary Material. Further inquiries can be directed to the corresponding authors.

## Supplementary Materials

The following supporting information can be downloaded at: https://www.mdpi.com/article/10.3390/cimb48060543/s1.

## Abbreviations

Full English Term	English Abbreviation
4′,6-diamidino-2-phenylindole	DAPI
Acute lung injury	ALI
Acute respiratory distress syndrome	ARDS
Alternatively activated macrophages	M2
Alveolar macrophages	AMs
Antioxidant response element	ARE
Beta-Actin	β-Actin
Bicinchoninic acid assay	BCA
Bone marrow-derived macrophages	BMDM
Bovine serum albumin	BSA
Catalase	CAT
CD11 antigen-like family member B	CD11B
Chronic obstructive pulmonary disease	COPD
Classically activated macrophages	M1
Electrochemiluminescence	ECL
Fetal bovine serum	FBS
Forkhead box O 1	FOXO1
Glutathione	GSH
Heat shock protein	HSP
Heat shock protein 60	HSP60
Heat shock protein 70	HSP70
Heat shock protein 90	HSP90
Heme pxygenase-1	HO-1
Horseradish peroxidasse	HRP
ImmunoglobulinG	Ig-G
Inducible nitric oxide synthase	iNOS
Interferon G=gamma	IFN-γ
Interleukin 1	IL-1
Interleukin-1β	IL-1β
Interleukin-6	IL-6
Ionized calcium binding adapter molecule 1	IBA1
Kelch-like ECH associated protein 1	Keap1
Lipopolysaccharides	LPS
Malondialdehyde	MDA
NF-κB p65	NF-κB RelA
Nicotinamide adenine dinucleotide	NAD+
Nitric oxide	NO
Nuclear factor erythroid 2-related Factor 2	NRF2
Phosphate buffered solution	PBST
Polyvinylidene fluoride	PVDF
Quantitative real-time PCR	qPCR
Reactive oxygen species	ROS
*Sirt2* knockout	*Sirt2*−/−
*Sirt2* knockout cold stimulation group	KO-Cold
*Sirt2* knockout room temperature group	KO-Control
Sirtuin2	SIRT2
Sodium dodecyl sulfate-polyacrylamide gel electrophoresis	SDS-PAGE
Superoxide dismutase	SOD1
Tris-buffered saline	TBST
Tumor necrosis factor alpha	TNF-α
Wild-type	WT
Wild-type cold stimulation group	WT-Cold
Wild-type room temperature group	WT-Control
