# Supplementary material for: SIRT2 Alleviates Chronic Cold Stress-Induced Lung Injury by Regulating Lung Macrophage M1 Polarization
Source: Curr Issues Mol Biol. 2026 May 22;48(6):543. doi: 10.3390/cimb48060543 (PMC13297586; doi:10.3390/cimb48060543)
Supplement: Supplementary file 1 [file cimb-48-00543-s001.zip › Supplementary Method.pdf]

Supplementary Method: Histopathological Lung Injury ScoringThe severity of lung injury was quantified using a modified semi-quantitative scoring system. For each lung section, five random fields were captured at 200× magnification. Each field was graded by two independent researchers on a scale from 0 to 4 based on the following criteria:

1. Alveolar septal thickening: assessed the degree of interstitial expansion.

2. Inflammatory infiltration: assessed the density of leukocytes in the alveolar and interstitial spaces.

3.Congestion and hemorrhage: assessed capillary dilation and erythrocyte leakage.The final lung injury score (LIS) for each section was calculated as the mean of these parameters.

Table S1. Semi-quantitative scores of lung injury in different experimental groups.

Group	Alveolar Septal Thickening	Inflammatory Infiltration	Congestion / Hemorrhage
WT-Control	1	1	0
Sirt2$^{-/-}$-Control	2	2	1
WT-Cold	4	4	3
Sirt2$^{-/-}$-Cold	3	3	2
